# BRIDGE pilot study: a bilateral regulatory investigation of data governance and exchange

**DOI:** 10.1038/s41746-025-02322-6

**Published:** 2026-02-17

**Authors:** Helen X. Hou, Tom Bisson, Sophia M. Leiss, Julia Thierauf, Ariel D. Stern, Hendrik Strobelt, Felix Nensa, Alena Buyx, Katharina M. Huster, Kira Furlano, Zisis Kozlakidis, Sachin Gupta, Danko Kostadinov, Peter Boor, Anna Slagman, Thorsten Tjardes, Pierre Cholet, Nick K. Schneider, Thorsten Schlomm, Saskia Biskup, Rainer Röhrig, Fruzsina Molnár-Gábor, Uta Schmidt-Straßburger, Katharina Ladewig, Marcel Weigand, Daniel Pinto dos Santos, Jason M. Johnson, Toralf Kirsten, Eric Sutherland, Norman Zerbe, Albert Hofman, Ralf Heyder, Georg Schmidt, Jochen K. Lennerz

**Affiliations:** 1 Department of Radiation Oncology, TUM University Hospital, Technical University of Munich, Munich, Germany; 2 Department of Pathology, Massachusetts General Hospital and Harvard Medical School, Boston, MA USA; 3 Charité—Universitätsmedizin Berlin, corporate member of Freie Universität Berlin, Humboldt-Universität zu Berlin, Institut für Medizinische Informatik, Berlin, Germany; 4 Charité—Universitätsmedizin Berlin, corporate member of Freie Universität Berlin, Humboldt-Universität zu Berlin, Institut für Pathologie, Berlin, Germany; 5 Department of Clinical Neuroscience, Institute of Neuroscience and Physiology, Sahlgrenska Academy, University of Gothenburg, Gothenburg, Sweden; 6 Foundation Medicine Inc, Cambridge, MA USA; 7 Digital Health Cluster, Hasso-Plattner Institute, University of Potsdam, Potsdam, Germany; 8 Harvard Business School Technology and Operations Management, Boston, MA USA; 9 Harvard-MIT Center for Regulatory Science, Boston, MA USA; 10 IBM Research/MIT-IBM AI Lab, Cambridge, MA USA; 11 Institute of Diagnostic and Interventional Radiology and Neuroradiology, University Hospital Essen, Essen, Germany; 12 Institute for Artificial Intelligence in Medicine (IKIM), University Hospital Essen, Essen, Germany; 13 Institute of History and Ethics in Medicine, Technical University of Munich, Munich, Germany; 14 German Ethics Council, Berlin, Germany; 15 Ethics Committee, Technical University Munich, Munich, Germany; 16 Charité—Universitätsmedizin Berlin, corporate member of Freie Universität Berlin, Humboldt-Universität zu Berlin, Klinik für Urologie, Berlin, Germany; 17 International Agency for Research on Cancer, World Health Organization, Lyon, France; 18 American Society for Clinical Pathology, Chicago, IL USA; 19 Colorectal and General Surgery, Princess Royal University Hospital, King’s College Hospital NHS Foundation Trust, London, United Kingdom; 20 Institute of Pathology, University Clinic Aachen, RWTH Aachen University, Aachen, Germany; 21 Charité - Universitätsmedizin Berlin, corporate member of Freie Universität Berlin, Humboldt-Universität zu Berlin, Notfallmedizinische Versorgungsforschung, Berlin, Germany; 22 Department of Trauma and Orthopedic Surgery, University of Witten/Herdecke, Cologne, Germany; 23 Decentriq, Berlin, Germany; 24 German Federal Ministry of Health, Berlin, Germany; 25 CeGaT und Zentrum für Humangenetik Tübingen, Tuebingen, Germany; 26 Institute of Medical Informatics, University Hospital RWTH Aachen, Aachen, Germany; 27 TMF—Technology and Methodology Plattform for Networked Medical Research e.V., Berlin, Germany; 28 Bio Quant / Faculty of Law, Heidelberg University, Heidelberg, Germany; 29 Master Online Study Program Advanced Oncology Ulm University Medical Faculty Division of Learning and Teaching, Ulm, Germany; 30 Center for Artificial Intelligence in Public Health Research, Robert Koch Institute, Berlin, Germany; 31 Department of Radiology, University Medical Center Mainz, Mainz, Germany; 32 Dana-Farber Cancer Institute, Boston, MA USA; 33 Institute for Medical Informatics, Statistics and Epidemiology, Leipzig University, Leipzig, Germany; 34 Dept. Medical Data Science, University of Leipzig Medical Center, Leipzig, Germany; 35 Organization for Economic Co-operation and Development (OECD), Paris, France; 36 Department of Epidemiology, Harvard T.H. Chan School of Public Health, Boston, MA USA; 37 Charité—Universitätsmedizin Berlin, Corporate member of Freie Universität Berlin and Humboldt-Universität zu Berlin, Network of University Medicine Coordination Office, Berlin, Germany; 38 Department of Internal Medicine I, TUM University Hospital, Technical University of Munich, Munich, Germany; 39 BostonGene, Waltham, MA USA

**Keywords:** Medical research, Risk factors

## Abstract

National privacy laws diverge between the European Union and United States, hindering transatlantic health data exchange and slowing AI-driven medical innovation. In response, the German Ministry of Health launched the pre-competitive *Data for Health initiative*, leading to the BRIDGE Pilot Study (2023–2025), a researcher-led effort to address this regulatory and legal gap. Using a mixed-methods approach, including structured surveys (*n* = 56 expert responses), ranking of steps via *relative importance indexing*, and 4 Delphi meetings, experts co-developed a practical framework composed of 30 steps in 3 consecutive phases for legally compliant and technically interoperable EU-US health data collaboration. The framework emphasizes early data protection assessments, secure transfer protocols, and iterative governance checks. The final consensus framework provides a stepwise guide to navigate regulatory and legal complexities and operationalize cross-border research. Ongoing input from researchers and stakeholders will help ensure the framework remains adaptable and provides a clear, scalable foundation for cross-border health data exchange.

## Introduction

Amidst a growing emphasis on domestic priorities^1,2^, divergences in healthcare policy are growing. The European Union (EU) and the United States (US) operate under distinct legal frameworks^3–5^. In the EU, the right to data protection is recognized as a fundamental right (Article 8, Charter of Fundamental Rights (CFR) of the EU), related to - but distinct from the right to privacy (Article 7, CFR of the EU)^3^. This status as a fundamental right shapes all subsequent legislation, including the General Data Protection Regulation (GDPR)^6^, the Artificial Intelligence (AI) Act^7^ and the recently adopted governance framework entitled *European Health Data Space* (EHDS)^8^. In contrast, the US Constitution does not establish data protection as a fundamental right^3^. Privacy protections are instead implemented through statutory law, most notably the Health Insurance Portability and Accountability Act (HIPAA^9^), evolving state-level privacy laws^10^ (e.g., California Consumer Privacy Act^11^, Colorado Privacy Act^12^) as well as ongoing attempts to craft additional federal legislation (e.g. *American Data Privacy and Protection Act* ADDPA^13^). Although both jurisdictions uphold patient privacy and promote ethical data use, their definitions of key terms (e.g., identifiability, pseudonymization, and the legal basis for data processing) remain distinct (Table 1). These structural and normative differences illustrate a current state of misalignment.

**Table 1:** Key Terminological Differences in EU–US Health Data Governance

Selected Terms	United States (HIPAA/ State Privacy Level)	European Union (GDPR/EHDS)	Implications for regulatory interoperability and/or data exchange
Identifiability	Narrow definition: “protected health information” (PHI) includes 18 specific identifiers under HIPAA	Broadly defined: includes any data that can be linked to an individual, even indirectly	EU considers more datasets as identifiable, triggering stricter requirements
Pseudonymization	Often treated as de-identified if re-identification risk is “very small”	A privacy-enhancing technique; still considered personal data under GDPR	US may treat pseudonymized data as non-PHI, creating legal asymmetry
Anonymization	Not explicitly defined in HIPAA; relies on “expert **determination**” or safe harbor criteria	Must be irreversible; anonymized data is no longer subject to GDPR	EU rarely accepts US anonymization techniques as compliant
Legal Basis for Processing of Health Data	Use and disclosure of PHI is generally prohibited unless permitted under the HIPAA Privacy Rule, e.g., for treatment, payment, healthcare operations, or certain public health, research, and legal purposes without individual authorization.	Processing of health data is generally prohibited under the GDPR unless a valid legal basis applies (Art. 6) and one of the specific conditions for processing special categories of data is met (Art. 2), i.e., explicit consent, provision of healthcare, reasons of public interest, or scientific research with appropriate safeguards	Fundamental legal logic differs; EU consent or additional national or Union law often required when US does not
Regulatory Focus and Governance Model	Institution-centered: regulates duties and permitted uses of covered entities and business associates	Individual-centered: defines rights of data subjects and lawful bases for controllers/processors	EU consent or additional law often required where US law relies on institutional authorization
Data Subject Rights	More limited: access and amendment under HIPAA; broader rights emerging under state laws (e.g. CCPA)	Broad: information, access, rectification, erasure, restriction, objection, portability	EU mandates rights incompatible with some US research workflows
Data Transfer Mechanisms	Relies on organizational compliance and contractual assurances	Requires adequacy decisions, SCCs, or other safeguards; DPF (2023) currently still valid	Post-Schrems II scrutiny makes EU–US transfers legally fragile

*CCPA* California Consumer Privacy Act, *DPF* EU–U.S. Data Privacy Framework, *EHDS* European Health Data Space, *EU* European Union; *HIPAA* Health Insurance Portability and Accountability Act, *PHI* Protected Health Information, *SCC* Standard Contractual Clauses, *US* United States.

Simultaneously, the AI revolution in medicine and public health depends on large, diverse, and high-quality datasets, raising critical concerns about access, equity, and responsible data governance^14–16^. Foundational principles such as individual rights, responsible use, and ethical guardrails are converging^17^, yet the operationalization of these ideals across jurisdictions lag^18^; particularly in healthcare. Transatlantic collaboration now sits at a critical juncture: constrained by regulatory and legal complexities yet driven by a shared imperative to accelerate therapeutic development, streamline health system operations, foster innovation, and improve the quality, efficiency, and patient-centricity of care through broader data access^19^. The adopted EHDS framework now offers a path forward for enabling secure, lawful data transfers between public institutions^8,19^ in Europe, whereas the *Trusted Exchange Framework and Common Agreement* (TEFCA)^20^ provides the backbone for nationwide health-information exchange in the United States.

To date, there is no practical instruction or ‘playbook’ that defines how to conduct legally compliant, ethically sound, and interoperable transatlantic health data exchange. Recognizing this, the German Federal Ministry of Health and US partners launched the precompetitive *Data for Health Initiative (*www.dfh23.de^21^*)*, bringing together researchers and policymakers to identify barriers and solutions. One outcome of this initiative is the BRIDGE Pilot Study (*Bilateral Regulatory Investigation of Data Governance and Exchange*; 2023–2025). Launched by researchers for researchers, this initiative defines the practical steps required for effective EU-US health data sharing. Here, we present an initial consensus-based framework to guide transatlantic collaborations through a structured, expert-informed approach. The larger context is to enable responsible, global-scale health data collaboration that accelerates research and improves care.

## Results

### Initial framework

**Fig. 1:**
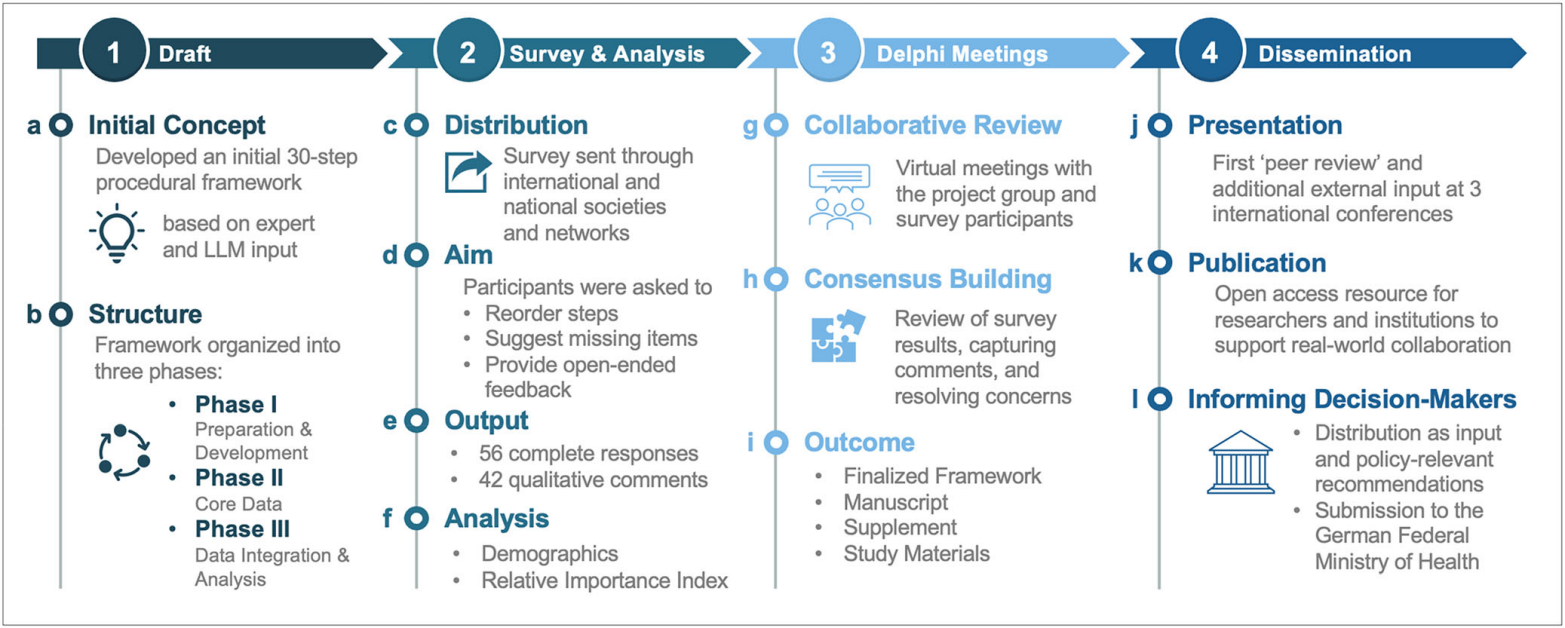
Project concept. The figure outlines the four components of the study. (1) Draft: An initial 30-step framework (a) was created based on expert and large language model (LLM) input and structured into three phases (b). (2) Survey & Analysis: The framework was evaluated through a survey (c), allowing reordering of steps and qualitative feedback (d). In total (e), 56 complete responses and 42 open-ended comments were analyzed (f). (3) Delphi Meetings: Iterative virtual meetings enabled collaborative review (g), refinement, and consensus building (h) toward the finalized framework (i). (4) Dissemination: The refined framework was presented at three international conferences (j), prepared for open-access publication (k), and submitted to the German Federal Ministry of Health as input for policy-relevant recommendations (l).

The drafting process resulted in a 30-step procedural framework (Fig. 1a and Table S1). The framework was structured into three phases that, in principle, reflect the progression of any research study: *Phase I:* preparation and development, *Phase II:* core data activities, and *Phase III:* data integration and analysis (Fig. 1b). This initial draft served as the foundation for iterative refinement through expert input.

### Survey

The survey was distributed through multiple national and international societies and institutional networks to ensure broad representation. The full list of distributing societies is provided in Table S2. A total of 178 individuals accessed the survey. After exclusion of *n* = 122 partial responses, *n* = 56 complete responses formed the basis for our analysis. The survey was composed of three main parts: demographics, ranking of items, and comments/feedback.

### Demographics

**Fig. 2:**
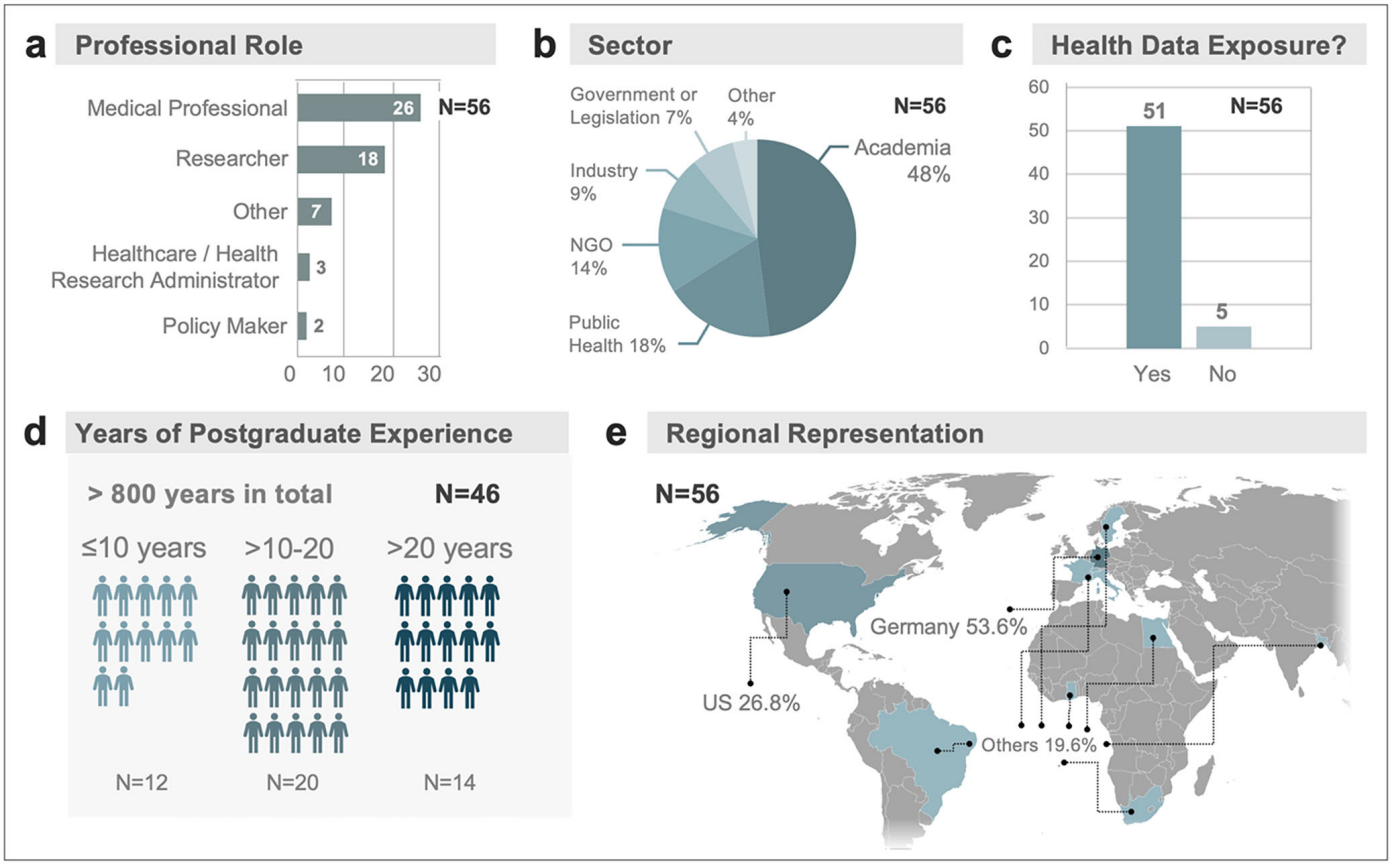
Survey participant demographics. Survey participants represented a range of (**a**) professional roles and (**b**) sectors, with the majority affiliated with academia. Most participants reported exposure to health data (**c**) and the majority had over a decade of postgraduate experience (**d**). Participants reported their workplace as based in Germany and the United States (**e**).

Demographic characteristics of the 56 participants (Fig. 2) shows the range of professional roles, with most participants identifying as medical professionals (*n* = 26, 46%) or researchers (*n* = 18, 32%; Fig. 2a). Nearly half were affiliated with academic institutions (*n* = 27, 48%), followed by the public health sector (*n* = 10, 18%) and non-governmental organizations (*n* = 8, 14%; Fig. 2b). Most participants reported involvement in health/patient data exchange (*n* = 51, 91.1%), particularly in research implementation and regulatory work (Fig. 2c). The participants had a cumulative postgraduate experience of over 800 years (Fig. 2d) with an average of 17.7 years, and a median of 17.5 years per participant (range 0–48 years). Following the 10,000 h rule^24^ at least *n* = 40 participants (71%) can be considered experts in their domain. Over half were based in Germany (*n* = 30, 53.6%), followed by the US (*n* = 15, 26.8%) and other countries (*n* = 11, 19.6% Fig. 2e). Although internationally distributed, all participants have direct experience in either the German/EU or US research and regulatory systems, ensuring that the framework’s EU–US focus is professionally grounded and relevant.

### Ranking of items

**Fig. 3:**
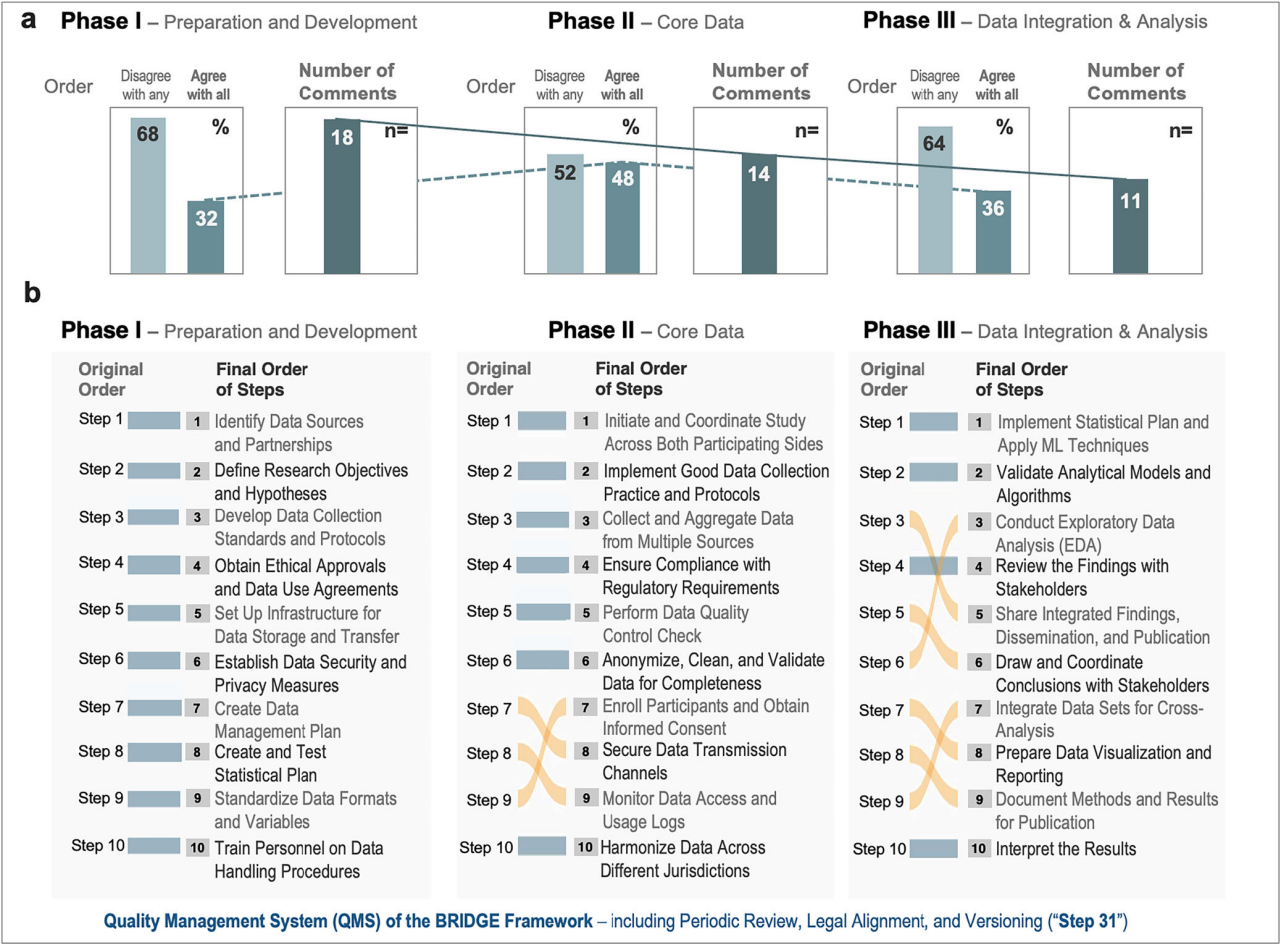
Survey results on step sequence across three project phases. **a** Bar charts indicate the levels of agreement and disagreement with the originally proposed step order, along with the number of comments received for each phase. **b** Based on survey input, the original steps were re-ordered within each phase to reflect a consensus-driven final sequence. The revised framework integrates these adjustments across all three phases.

Participants reviewed and either confirmed or reordered the original 10 procedural steps within each of the three framework phases (Fig. 3; Table S3). In the *Preparation and Development Phase*, *n* = 18 out of 56 participants (32%) agreed with the original order, *n* = 38 (68%) reordered at least one item. In the *Core Data Phase*, *n* = 27 participants (48%) retained the proposed order, *n* = 29 (52%) made changes. In the *Data Integration and Analysis Phase*, *n* = 20 participants (36%) agreed with the initial sequence, *n* = 36 (64%) made modifications (Fig. 3a). Across phases, *the Preparation and Development Phase* retained the original sequence (Fig. 3b). The *Core Data Phase* showed relatively minor reordering concentrated in lower-ranked steps (i.e., steps II: 7, 8 and 9). In contrast, the *Data Integration and Analysis Phase* showed substantial shifts in sequence, reflecting greater variability in later stage analytical workflows (i.e., steps III: 3-9). The final order of steps was established by the RII for each phase (see Table S4).

### Comments

Participants were invited to suggest missing steps and provide open-ended feedback. This generated 42 written comments for the three phases (Figs. 1e, 3a). The following summary reflects key feedback points (for a full list see Table S5–S7).

In the *Preparation & Development Phase (Phase I*; Table S5*)*, there was strong support for early planning of data protection impact assessments, consultation with data protection authorities, and consideration of interoperability standards such as Fast Healthcare Interoperability Resources (FHIR). One participant advocated for a two-step ethical approval process in transatlantic collaborations. Another proposed appointing a Chief Data Officer to coordinate multi-step compliance and communication.

In the *Core Data Phase (Phase II*; Table S6*)*, participants highlighted the need for secure data transmission, early testing to identify process vulnerabilities, and parallelization of steps to optimize timelines. Several participants emphasized the integration of regular feedback loops and quality checks.

In the *Data Integration and Analysis Phase (Phase III*; Table S7*)*, there was repeated emphasis on the importance of conducting exploratory data analysis earlier in the sequence to ensure data readiness. Participants also commented on model validation, especially in evolving datasets, and promoted the use of data visualization and re-validation strategies. Additional suggestions included planning for data archiving, and dissemination strategies to non-technical stakeholders.

The number of steps participants chose to *move* increased across the survey phases (Fig. 3b), whereas the number of written *comments* decreased (Phase I: 18, Phase II: 14, Phase III: 11; Fig. 3a). Notably, participants already made substantial rearrangements in Phase I, as reflected by the rank-sum of deviations (n = 229 in Phase I vs. n = 193 in Phase II and n = 272 in Phase III). Despite these shifts, the final RII-based order remained essentially unchanged. Similarly, Phase I showed that 68% of participants disagreed with at least one step (Fig. 3a); however, these alterations did not manifest in the RII-based final order either.

### Delphi meetings

We hosted four modified Delphi meetings, with *n* = 22, *n* = 14, *n* = 9, and *n* = 8 attendees, respectively. Of note, we recruited *n* = 11 participants through the survey (opt-in question). Attendance and session outcomes were documented for each meeting (Table S8). The meetings addressed several topics: participant demographics, item lists per phase (I, II, III) alongside the comments, as well as review and discussion of the finalized RII-based framework, manuscript, tables, and figures. Many comments were discussed in depth during the Delphi meetings and informed the final refinement of the framework. For example, one suggestion during a meeting was to compile the specific list of stakeholders responsible for each step in the final framework, whether cross-coordination across EU-US would be necessary, and their relation to specific study types. This table was constructed, reviewed and is provided as Table S9. During several meetings, data integrity was highlighted as a core concept, particularly the ALCOA principle (Table S10). One additional suggestion was to initiate the collection of relevant web resources; now provided as Table S11. Key elements from these collaborative review sessions helped shape this publication, tackled contextualization of our results, and emphasized the need for further tailoring based on specific study topics. We also incorporated feedback from presentations at three international conferences that collectively re-emphasized the need for a practical framework.

## Discussion

Here, we report the results of the BRIDGE Pilot Study, a transatlantic effort to co-develop a stepwise, consensus-based framework for legally compliant, ethically sound, and technically interoperable health data exchange between the EU and the US. This study is the first to systematically map the procedural requirements for cross-border data collaboration using a mixed-methods approach that combines expert surveys, procedural step-ranking, and iterative Delphi consensus. By integrating diverse stakeholder perspectives and resolving conflicting priorities, the study delivers a practical, adaptable blueprint that addresses long-standing and evolving regulatory and operational gaps. The resulting 30-step framework reflects not only current legal requirements but also offers a scalable structure for future policy alignment and research implementation.

Cross-border access to high-quality health data is becoming increasingly essential for enabling AI-driven research, accelerating therapeutic development, and responding to global public health challenges; yet no established roadmap exists for how to do this in a compliant, ethical, and interoperable manner. The context of transatlantic health data sharing is shaped by three interrelated forces: (1) the growing reliance on AI in medicine and public health, (2) the persistent legal misalignment and different operational implementations between the EU and US, (3) and the direct experience of researchers navigating these complexities. Recent developments underscore the importance of similar projects^19,21,25^. Practical interoperability continues to be limited by regulatory and legal divergence. The GDPR and HIPAA, in particular, differ in their legal bases for processing, scope of protections, and definitions of identifiable data^26–28^. These differences have previously disrupted transatlantic collaboration, most notably after the *Schrems II* ruling^29,30^, which invalidated the EU-US “Privacy Shield” in 2020 due to concerns over US surveillance practices. The 2023 EU-US Data Privacy Framework (DPF)^31^ was introduced to address these concerns, and as of 2023, was approved by the European Commission as a valid adequacy decision^32^. However, the DPF applies only to private-sector entities and excludes public institutions such as universities and government agencies processing health data^33^. Moreover, given the history of prior adequacy frameworks being invalidated, questions remain about the long-term stability of the DPF and its suitability for research collaborations. In the EU, the adoption of the EHDS Regulation^25,34^ in March 2025 aims to standardize the use and exchange of electronic health data across member states, enhancing individuals’ access and control over their personal data while facilitating reuse for *inter alia* research and innovation^35^. The EHDS introduces a sector-specific model that may enable health-related data sharing across borders, potentially sidestepping broader adequacy debates between the EU and US^19^. However, as highlighted by Lalova-Spinks et al., the success of this approach will depend on several factors: (a) whether the European Commission, through its implementing acts, provides a clear pathway for the applicable GDPR transfer mechanisms; (b) whether US authorities are willing to participate in the EHDS infrastructure by designating a national contact point; and (c) whether sovereign immunity concerns can be addressed through this contact point^19^. In addition, secondary use of health data through the common ‘HealthData@EU’ governance infrastructure^36^ will only be applicable as of March 2029 and other countries will only be able to participate as of March 2035. Concurrently, the US Department of Health and Human Services (HHS) has proposed significant updates to the HIPAA Security Rule to strengthen^37^ cybersecurity for electronic Protected Health Information (ePHI)^38^. These changes require covered entities to implement encryption, multifactor authentication, and updated risk assessments. While intended to improve data security, these reforms may further complicate compliance and delay or hinder coordination in cross-border data exchange.

Given these dynamics, the BRIDGE Pilot Study delivers more than a procedural framework. We offer a shared foundation for trust-building and a “playbook” for operational alignment in a conceptually distinct regulatory landscape (Table 1). By elevating practitioner insights into a structured, consensus-driven model, the study helps translate abstract legal principles into actionable guidance. Its pragmatic orientation fills a critical void between high-level policy ambition and the day-to-day realities of international research collaboration. We are also sharing this framework as input for policymakers, regulators, and legislators: i.e., as concrete bottom-up insights from researchers (Table S12). By sharing results of initiatives such as BRIDGE, policymakers can gain direct insight into the practical challenges and solutions of transatlantic health data sharing. Although formal adoption in Germany and the US has not yet occurred, these early efforts lay the groundwork for constructive dialogue and eventual regulatory integration (i.e., top-down; Table S12). Policymakers can use this foundation to develop legally compliant and operationally feasible policies aligned with real-world research practices. The *America’s AI Action Plan*^39^ similarly encourages regulators to evaluate how existing law applies to emerging technologies. Sustained dialogue between policymakers and researchers remains essential to resolve legal and regulatory barriers. Strengthening this engagement may involve integrating policymaker into transatlantic working groups, highlighting the policy relevance of research outputs, and fostering trusted pre-competitive collaborative research communities.

We note that the concerns raised by legal departments extend beyond our specific approach, particularly regarding engagement with the U.S., where the lack of effective legal remedies and GDPR restrictions on data sharing without valid consent remain unresolved issues. While Hussein et al.^25^, and others^40–42^ have provided toolkits for specific research questions or use cases, we are not aware of a previously published general framework. Our study creates space for bilateral alignment between two important regulatory ecosystems (Germany and the US) while offering a template that can be adapted across jurisdictions. We acknowledge that Germany’s federal structure leads to regional variation in the implementation of EU and national laws; nonetheless, our framework offers procedural guidance designed to be adaptable to local and institutional settings. As legal frameworks evolve and digital health infrastructures mature, such practical instruments will be essential to ensure that compliance does not become a barrier to progress, but a channel through which global health data collaboration can responsibly scale.

Several limitations of this study must be acknowledged. First, the framework was developed through expert consensus and has not yet been tested in a live cross-border data exchange, meaning its practical utility in specific institutional or national contexts remains to be validated. Applicability will vary across settings due to local legal and infrastructural conditions, underscoring the need for adaptation and testing. Thus, a key limitation of any new general framework, including BRIDGE, is the initial lack of evidence for its practical applicability. Second, the survey relied on voluntary participation, introducing potential selection bias and limiting the generalizability of findings beyond the primarily German and US-based respondent pool. Third, the survey design required participants to select only one professional role, which may not fully capture individuals concurrent or prior roles and thus likely underrepresents the diversity of professions. Furthermore, the increasing number of step changes over the course of the survey (Fig. 3b) may reflect growing familiarization with the sorting interface, while the decline in comments suggests emerging survey fatigue^43^. In addition, it seems counterintuitive that 68% of participants in Phase I disagreed with the initial order while the final order remained unchanged. However, the rank-sum of deviations indicates that participants did meaningfully reorder items–even though these individual changes did not substantially alter the final order based on the relative importance index (Fig. 3b Phase I). While this may suggest a potential limitation or bias in the relative importance index, we provide the raw data to enable alternative analytical approaches. Fourth, while the Delphi process enabled iterative refinement, it was conducted virtually and without formal anonymity, which may have influenced group dynamics or constrained dissenting perspectives. Fifth, although there are emerging technical approaches to data federation^44^, synthetic data generation^45^, and privacy-preserving computation^46^, these were not the focus of this pilot. We intentionally worked under the premise that, in some research scenarios (e.g., event traceability or regulatory decision-making), the exchange of actual individual-level health data remains necessary. Finally, the framework reflects the regulatory environment as of mid-2025; given the rapid pace of legal and technological change (particularly with respect to EHDS, HIPAA reforms, and AI governance), ongoing revision will be necessary to maintain its relevance and applicability.

Looking ahead, the BRIDGE framework can serve as a living tool to support evolving policy efforts and research practices. As institutions prepare for the operationalization of the EHDS and respond to shifting US data protection standards, including recent executive actions that signal a more restrictive stance on certain cross-border data flows, there is a critical opportunity and growing need to pilot and refine the framework in real-world settings. Its application will also require both procedural and technical resources, including integration into existing (local) governance structures, secure infrastructures, and standardized templates. To support this, we provide a table with useful resources in the supplement (Table S11). In parallel, we are working on concrete transatlantic data exchange use case projects that apply elements of the framework to test and refine its practical applicability. Future work should further validate the framework across diverse legal, technical, and cultural contexts, while promoting dialogue and shared infrastructure development^19^. To strengthen the framework’s responsiveness to evolving legal and regulatory conditions, we explicitly incorporated adaptability into its structure (Table S13; Modifiable Bridge Framework Document).

The presented BRIDGE framework also includes a proposed quality management system (QMS; Fig. 3 Step 31). Briefly, the QMS is organized in 7 modules to orchestrate periodic review, legal alignment, and versioning (Table S13). We also provide additional considerations and practical example questions for local refinement. Adaptability is thereby operationalized through scheduled review triggers, branching logic for alternative regulatory conditions, and explicit integration of legal–policy expertise at key decision points. These additions ensure that the framework can evolve as the EHDS secondary acts are adopted, as US Executive Orders or federal privacy initiatives emerge, and as institutional interpretation of GDPR/HIPAA requirements shifts over time. As a result, and if resource permit, the BRIDGE framework can transition from a static proposal to a living procedural tool with clearly defined mechanisms for continuous alignment with regulatory change. A modifiable version of the BRIDGE framework is provided as a supplement.

The BRIDGE Pilot Study delivers a consensus-based, stepwise and adaptable framework intended to support ethical, legally sound, and technically feasible health data exchange between the EU and the US. While its ultimate applicability will depend on further testing and validation, it provides timely procedural guidance amidst regulatory uncertainty and offers a practical foundation for researchers and policymakers to build scalable, trustworthy international data collaborations.

## Methods

### Study design

This pilot study applied a mixed methods design to develop a framework for compliant transatlantic health data exchange. The study is structured into four key sections:

(1) Draft, (2) Survey and Analysis, (3) Delphi Meetings, and (4) Dissemination.

### Draft

The initial framework (Fig. 1a,b) was developed based on the professional experience of the authors, including clinicians, policymakers, researchers, and experts in medical law and medical informatics from both the EU and the US. To enhance clarity and consistency, we also used a large language model (LLM; OpenAI GPT-4.0) for proofreading and cross-checking.

### Survey and analysis

A structured survey was created using Qualtrics (Qualtrics, Provo, UT) and distributed through national and international societies and institutional networks (Fig. 1c). The survey was conducted between Nov 8^th^, 2024 and Apr 8th, 2025. The primary aim of the survey was to gather expert input by having participants reorder 30 procedural steps, suggest missing elements, and provide open-ended feedback based on their practical experience (Fig. 1d). In addition, we provided an opt-in page to join subsequent meetings (Delphi process; see below). A total of 56 complete responses and 42 qualitative comments were collected (Fig. 1e).

Analysis of demographic variables included gender, country of residence, professional role, employment sector, years of postgraduate experience, board certification status (if applicable), and involvement in health data governance; all data were analyzed using Microsoft Excel for macOS (version 16.94). Frequencies and percentages were reported for categorical variables (Fig. 2). We distinguished between agreement (for all steps) and disagreement (with at least one step) for calculation of overall agreement/disagreement percentages. We defined the rank-sum of deviations (from original order) as the total absolute difference in positions between each item’s original rank and its new rank after participant reordering–summed across all items (and participants). This provides a single numeric summary of how much a cohorts ranking deviated from the original order. During the analysis we assessed the relative priority of each procedural step using the Relative Importance Index (RII) calculated from participant rankings. RII was computed using the formula RII = Σ(W × X) / (A × N), where W is the weight assigned to each rank (inversely proportional to position, with rank 1 receiving weight 10), X is the number of respondents assigning that rank, A is the highest possible rank (10), and N is the total number of respondents. This normalization results in a score between 0 and 1, where values closer to 1 indicate steps generally placed earlier in the process (Fig. 1f).

### Delphi meetings

Survey results were reviewed through a series of four online meetings (Mar 21st, 2025; Apr 14th, 2025; May 5th, 2025; Jun 4th, 2025), using a modified Delphi method to refine the procedural framework and reach expert consensus (Fig. 1g). The Delphi approach is particularly well suited for contexts where definitive evidence is limited, and expert opinion plays a critical role. Meetings brought together members of the initial project group and survey participants, enabling structured discussions of conflicting priorities and qualitative feedback (Fig. 1h). We followed established reporting guidance for the Delphi process^22,23^ and documented the number of participants, activities conducted, and discussion outcomes at each stage. Each session was recorded, independently reviewed by two authors (HXH and JKL), and all expert comments and clarifications were tracked and circulated to the authors, forming the basis for iterative refinement. Relevant input was collected by e-mail and incorporated in subsequent meetings, the manuscript and, when applicable, accompanying study documentation (Fig. 1i). The framework was shaped through an open, iterative process in which drafts were circulated to policy and legal experts at international meetings and through individual consultations. Their comments were transparently logged, reviewed, and incorporated into successive revisions, ensuring that specialist expertise is embedded in the final version.

### Dissemination and feedback

The study was disseminated through multiple channels to maximize visibility and impact. This included presentation at three international conferences (Fig. 1j). Specifically, the German American Conference 2024 (Boston, Nov 14–16th 2024), the Robert Koch Institute (RKI) AI in Public Health Research Symposium (Berlin, May 14–15th 2025), and the European Congress of Digital Pathology (ECDP) 2025 (Barcelona, Jun 25–28th 2025). The agreed upon study design included an open access, peer-reviewed publication (Fig. 1k) and ultimately will entail sharing of the published results through the Data for Health community and associated stakeholder groups to support ongoing policy discussions and promote sustainable transatlantic collaboration (Fig. 1l).

### Ethics approval and consent to participate

This study collected survey data and expert input through a Delphi-style process. In accordance with applicable guidelines for minimal-risk, non-clinical online research, formal review by an ethics committee was not required. All participants were informed about the study purpose, voluntary participation, and data protection measures consistent with the General Data Protection Regulation (GDPR). Completion of the survey was taken as indication of informed consent. Specifically, after completion, participants could voluntarily provide their name and email address if they wished to be included in subsequent Delphi meetings; this information was collected separately from survey responses to ensure confidentiality.

## Supplementary information

supplementary tables

supplementary information

## Data Availability

All data generated or analyzed during this study are included in this published article and its supplementary information files. Recordings of Delphi meetings are available upon request.
