# Supplementary material for: BRIDGE pilot study: a bilateral regulatory investigation of data governance and exchange
Source: NPJ Digit Med. 2026 Feb 17;9:244. doi: 10.1038/s41746-025-02322-6 (PMC13022335; doi:10.1038/s41746-025-02322-6)
Supplement: Supplementary file 2 — supplementary information [file 41746_2025_2322_MOESM2_ESM.docx]

**BRIDGE Framework
Modifiable Core Steps Document (Draft Template)**

*A structured workflow for cross-border research readiness, data governance, and EU–US data transfer compliance.*


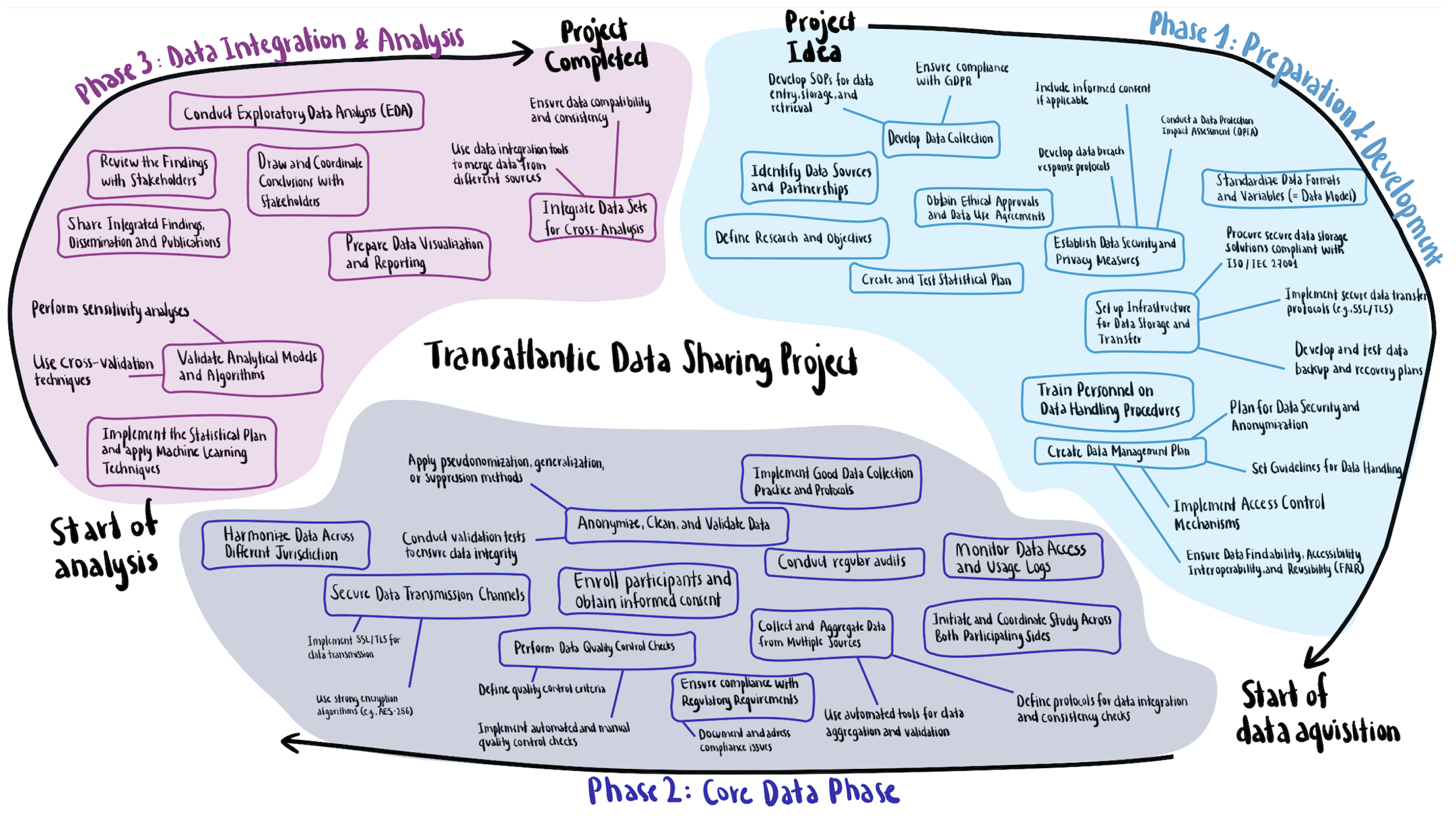


***The following document is composed of 4 sections***

*Page*

*Phase 1. Preparation and Development (Steps 1-10) 2*

*Phase 2. Core Data Phase (Steps 11-20) 3*

*Phase 3. Data Integration and Analysis (Steps 21-30) 4*

*Quality Management System related items (Step 31) 5*

**Phase 1 – Preparation & Development**

*Objective: Establish legal, technical, and procedural readiness before data collection begins.*

| **Step** | **Description** | | **Notes / Local Adaptation** |
| --- | --- | --- | --- |
| 1 | Identify Data Sources and Partnerships | |  |
| 2 | Define Research Objectives and Hypotheses | |  |
| 3 | Develop Data Collection Standards and Protocols | |  |
| 4 | Obtain Ethical Approvals and Data Use Agreements | |  |
| 5 | Set Up Infrastructure for Data Storage and Transfer | |  |
| 6 | Establish Data Security and Privacy Measures |  |  |
| 7 | Create Data Management Plan | |  |
| 8 | Create and Test Statistical Plan | |  |
| 9 | Standardize Data Formats and Variables | |  |
| 10 | Train Personnel on Data Handling Procedures | |  |

Notes:

**Phase 2 – Core Data Phase**

*Objective: Execute high-quality, compliant data collection, processing, and secure handling.*

| **Step** | **Description** | **Notes / Local Adaptation** |
| --- | --- | --- |
| 11 | Initiate and Coordinate Study Across Both Participating Sides |  |
| 12 | Implement Good Data Collection Practice and Protocols |  |
| 13 | Collect and Aggregate Data from Multiple Sources |  |
| 14 | Ensure Compliance with Regulatory Requirements |  |
| 15 | Perform Data Quality Control Check |  |
| 16 | Anonymize, Clean, and Validate Data for Completeness |  |
| 17 | Enroll Participants and Obtain Informed Consent |  |
| 18 | Secure Data Transmission Channels |  |
| 19 | Monitor Data Access and Usage Logs |  |
| 20 | Harmonize Data Across Different Jurisdictions |  |

Notes:

**Phase 3 – Data Integration & Analysis**

*Objective: Integrate, analyze, interpret, and responsibly disseminate cross-border, multi-source datasets.*

| **Step** | **Description** | **Notes / Local Adaptation** |
| --- | --- | --- |
| 21 | Implement Statistical Plan and Apply ML Techniques |  |
| 22 | Validate Analytical Models and Algorithms |  |
| 23 | Conduct Exploratory Data Analysis (EDA) |  |
| 24 | Review the Findings with Stakeholders |  |
| 25 | Share Integrated Findings, Dissemination, and Publication |  |
| 26 | Draw and Coordinate Conclusions with Stakeholders |  |
| 27 | Integrate Data Sets for Cross-Analysis |  |
| 28 | Prepare Data Visualization and Reporting |  |
| 29 | Document Methods and Results for Publication |  |
| 30 | Interpret the Results |  |

Notes:

**QMS documentation***Step 31: Ensuring Adaptability, Governance, and Continuous Alignment (details in Hou et al., Table S-13)* ***Module 1 — Versioning & Update Mechanism****Local accountable role(s): ______________________________________
Scheduled review frequency: ____________________________________
Trigger-event monitoring process (e.g., adequacy rulings, EO updates):Location of change-log / version-control documentation: __________________*

***Module 2 — Policy–Procedure Crosswalk****Responsible policy/legal reviewer: ________________________________
Process for updating crosswalk when laws change:
Legal sources referenced (GDPR/EHDS/HIPAA/DPF/state laws):__________________*

***Module 3 — Conditional Pathways****Decision authority for pathway selection: __________________________
Criteria for pathway choice (adequacy, SCCs, risk tier):Documentation method for chosen pathway: ________________________*

***Module 4 — Interdisciplinary Integration****Required disciplines represented (tick all that apply):
☐ Legal ☐ Regulatory ☐ Ethics ☐ Technical ☐ Clinical ☐ Data governance
Defined minimum competencies / roles:Review checkpoint timing (e.g., before transfer decision): __________________*

***Module 5 — Adaptability Triggers (Use-Case Adjustments)****Local triggers identified (e.g., policy changes, institutional reclassification):Process for detecting and applying triggers: ________________________*

***Module 6 — Resource Requirements & Feasibility****Minimum required resources available (Y/N):
DPO ____ Legal counsel ____ IT security ____ DPIA/TIA capability ____
Local limitations & mitigation plan: _______________________________*

*Selected mode: ☐ Full implementation ☐ Lightweight implementation*

***Module 7 — Procedural Guarantees & Governance****Framework owner / steward: ____________________________________
Responsible party for version releases: ____________________________
Compliance verification process: _________________________________*

*Record-keeping / audit documentation location: __________________*

***Summary of Local Adoption***

*Date of adoption: ______________________
Authorized signatory: ____________________
Next scheduled review: __________________*
